# Sheng Mai San protects H9C2 cells against hyperglycemia-induced apoptosis

**DOI:** 10.1186/s12906-019-2694-2

**Published:** 2019-11-12

**Authors:** Bing Pang, Li-Wei Shi, Li-juan Du, Yun-Chu Li, Mei-Zhen Zhang, Qing Ni

**Affiliations:** Department of Endocrinology, Guang’ anmen Hospital of China Academy of Chinese Medical Sciences, 6 floors of Inpatients building, 5 Beixiange Street, Xicheng District, Beijing, China

**Keywords:** Sheng Mai San, H9C2 cells, Apoptosis, Bcl-2/Bax signaling way, Fas/FasL signaling way

## Abstract

**Background:**

Sheng Mai San (SMS) has been proven to exhibit cardio-protective effects. This study aimed to explore the molecular mechanisms of SMS on hyperglycaemia (HG)-induced apoptosis in H9C2 cells.

**Methods:**

HG-induced H9C2 cells were established as the experimental model, and then treated with SMS at 25, 50, and 100 μg/mL. H9C2 cell viability and apoptosis were quantified using MTT and Annexin V-FITC assays, respectively. Furthermore, Bcl-2/Bax signalling pathway protein expression and Fas and FasL gene expression levels were quantified using western blotting and RT-PCR, respectively.

**Results:**

SMS treatments at 25, 50, 100 μg/mL significantly improved H9C2 cell viability and inhibited H9C2 cell apoptosis (*p* < 0.05). Compared to the HG group, SMS treatment at 25, 50, and 100 μg/mL significantly downregulated p53 and Bax expression and upregulated Bcl-2 expression (*p* < 0.05). Moreover, SMS treatment at 100 μg/mL significantly downregulated Fas and FasL expression level (*p* < 0.05) when compared to the HG group.

**Conclusion:**

SMS protects H9C2 cells from HG-induced apoptosis probably by downregulating p53 expression and upregulating the Bcl-2/Bax ratio. It may also be associated with the inhibition of the Fas/FasL signalling pathway.

## Background

Diabetes mellitus (DM) is on the verge of becoming a global epidemic. According to a report by the International Diabetes Federation, DM currently affects nearly 4250 million people [1]. In China, DM affects 10.9% of the population, which accounts for about a third of the diabetic patients worldwide [2]. Cardiovascular complications are the primary cause of disability and death due to DM. The medical expenses associated with diabetic macrovascular complications accounts for 80% of the total amount, making DM a huge economic burden on the society [3]. Therefore, it is imperative to prevent or delay the onset and development of diabetic cardiovascular complications. Diabetic cardiomyopathy (DCM), a major complication associated with DM, is defined as the dysfunction of the left ventricle in diabetic patients, in the absence of coronary artery disease or hypertension [4, 5]. It is initially characterized by left ventricular diastolic dysfunction and interstitial fibrosis, followed by systolic dysfunction and ejection fraction, and eventually resulting in heart failure [5–7]. Notably, cardiomyocyte apoptosis plays an important role in the pathophysiological mechanisms associated with DCM. There are two major mechanisms regulating this apoptosis. The first mechanism involves an intrinsic pathway, also called ‘the mitochondrion pathway’, such as the one regulating the B cell lymphoma/leukemia-2 (Bcl-2) protein family. The other apoptosis mechanism occurs via signaling by death receptor members, such as factor associated suicide (Fas)/factor associated suicide ligand (Fas-L) [8]. Hyperglyceamia activates the protein 53 (p53), and the renin-angiotensin system (RAS), resulting in the production of angiotensin II (Ang II), which leads to a decrease in Bcl-2 expression and an increase in Bcl-2 associated X protein (Bax) expression and, thus, plays an important role in promoting apoptosis [9, 10]. Bcl-2 and Bax are key proteins that regulate apoptosis [11]. P53 is a tumour suppressor gene that induces apoptosis by blocking cellular DNA damage repair [12]. Fas/FasL signaling is also crucial for cardiomyocyte apoptosis as it mainly regulates caspase-3 [13]. Currently, Western medicine is mainly focused on glycaemic control and treatment or prevention of risk factors associated with cardiovascular disease; however, these approaches do not fundamentally solve the problem of cardiac dysfunction [4, 14, 15].

Sheng Mai San (SMS) is a classical traditional Chinese formula containing the root of *Radix Ginseng* (Ren Shen), rootstock of *Radix Ophiopogonis* (Mai Dong), and dry ripe fruit of *Fructus Schisandrae* (Wu Wei Zi), which is recorded in the Yi Xue Qi Yuan, compiled by Yuan-su Zhang. It was mainly used for treating heart failure, myocardial ischemia, coronary heart disease, arrhythmia, myocarditis, and sick sinus syndrome [16–18]. We have earlier shown that SMS treatment alleviated myocardial damage and inhibited myocardial fibrosis in diabetic rats. SMS has also been proven to suppress cardiomyocyte apoptosis; however, its upstream mechanism is still unclear [19, 20]. Therefore, in this study, our aim was to explore the mechanisms underlying SMS activity with respect to cardiomyocyte apoptosis and provide new scientific evidence in favor of using traditional Chinese medicine to prevent DCM related damage.

## Methods

### Sheng Mai San standardisation

SMS was provided by Kangmei Pharmaceutical Co., Ltd. after adequate quality measurement. All the herbs were taken from the same batch. Decoctions were made at Guang’ anmen Hospital, China Academy of Chinese Medical Sciences, according to standard operating procedures. The major compounds in SMS were identified using high-performance liquid chromatography (HPLC; Waters 2695 HPLC system; Waters, CA, USA). A Luna® Omega Polar C18 analytical column (250 × 4.6 mm, 3.0 μm; Phenomenex, CA, USA) with a mobile phase that contained acetonitrile (A) and − 0.2% phosphoric acid acid in water (B) was used. The mobile phase gradient elution was programmed as follows: The mobile phase gradient elution was programmed as follows: 27% A (0–10 min), 27–38% A (10–12 min), 38% A (12–20 min), and 38–90% A (20–60 min); 73% B (0–10 min), 73–62% B (10–12 min), 62% B (12–20 min), and 62–10% B (20–60 min). The column temperature was maintained at 35 ∘C, the flow rate was set at 0.5 mL/min, and a detection wavelength of 203 nm was used. SMS was dissolved in double distilled water containing 0.05% dimethylsulfoxide (DMSO). The solution was centrifuged, filtered and disinfected using a syringe filter (specification: 13 mm nylon filter, 0.45 μm,100 pcs/pack), and preserved at − 20 ∘C for further experimentation [21].

### Cell culture and drug treatment

Rat embryonic cardiomyoblast-derived H9C2 cells were obtained from the Cell Culture Center of the Institute of Basic Medical Sciences, Chinese Academy of Medical Sciences (Beijing, China). The cells were starved in Dulbecco’s Modified Eagle Medium (DMEM) containing 10% FBS and 1% penicillin/ streptomycin and cultured in a humidified atmosphere containing 5% CO2 at 37 °C for 24 h till they reached 60–70% confluency. H9C2 cells were then cultured in different sets for 24 h in DMEM containing a) 5.5 mM normal glucose (N), b) 30 mM D-glucose (H), c) 30 mM D-glucose with 25 μg/mL of SMS (25), d) 30 mM D-glucose with 50 μg/mL of SMS (50), and e) 30 mM D-glucose with 100 μg/mL of SMS (100). The requisite glucose concentration for inducing HG was determined based on a previously published study [22].

### Cell viability analysis

H9C2 cell viability was detected via the 3-(4,5-Dimethylthiazol-2-yl)-2,5-diphenyltetrazoliumbromide (MTT) assay, for which the cells were maintained for 24 h. The cells were treated with SMS, following with, they were incubated with MTT solution (0.5 mg/mL) for 4 h at 37 °C. The supernatant was discarded, 110 μL of 0.05% DMSO was added to each well in a 96-well plate, and the cells were incubated for 10 min. Absorbance (OD value) was measured using a microplate reader at a wavelength of 490 nm. Percentage of reduced MTT was considered to represent the decrease in H9C2 cell viability.

### Cell apoptosis assay

H9C2 cell apoptosis was detected via Annexin-V fluorescein isothiocyanate/ propidium iodide (Annexin V-FITC/PI) staining. For this procedure, H9C2 cells were harvested using 0.05% trypsin, washed twice with cold phosphate buffered saline (PBS) (4 °C), and resuspended in 500 μg/mL of binding buffer at a concentration of 1 × 10^5^ cells/mL. The cells were then incubated with Annexin V-FITC (5 μg/mL) and PI (5 μg/mL) in the dark for 15 mins at room temperature.

### Cell-cycle analysis

H9C2 cells were cultured in DMEM for 24 h and then seeded at 4 × 10^5^ cells/well in a 6-well culture plate. SMS was added as described in the section “Cell culture and drug treatment”. After treatment, the cells were collected and washed twice with PBS solution. RNase A solution (100 μL) was added, and the cells were incubated for 30 min at 37 °C, followed with 70% ethanol and then fixed at 4 °C for 2 h overnight. Subsequently, the cells were washed with PBS to remove the ethanol. Finally, cells were stained with 400 μL PI and incubated for 30 min at room temperature, and cell staining was measured using flow cytometry. The results were analyzed using the Cell Quest software. Percentage of cells in the G1 phase, the S phase, and the G2 phase was analyzed.

### Western blotting

After SMS treatment, H9C2 cells were harvested, washed with cold phosphate-buffered saline (PBS), and incubated in radio immunoprecipitation assay (RIPA) buffer solusion (Solarbio, China) on ice. The total protein was then extracted from the cells and was quantified using a bicinchoninic acid (BCA) assay kit (Cwbiotech, China). Cell lysates were added to a loading buffer, separated using 12% sodium dodecyl sulfate-polyacrylaminde gel electrophoresis (SDS-PAGE), and transferred onto polyvinylidene fluoride (PVDF) membranes (Millipore, USA). The membranes were blocked using Tris-Buffered Saline (TBS) blocking buffer and incubated with primary antibodies (CellSignaling Technology, USA) overnight at 4 °C. The dilution ratios used were as follows: rabbit monoclonal antibodies against Bcl-2 (1: 1000) and Bax (1: 1000), mouse monoclonal antibody against p53 (1: 1000). After washing the membranes thrice with Tris-Buffered Saline Tween-20 (TBST), they were incubated with HRP-conjugated secondary antibodies (Jackson ImmunoResearch, USA) for 1 h. The dilution ratios used were as follows: goat anti-rabbit antibody (1: 1500) and goat anti-mouse antibody (1: 1500). A chemiluminescence enhancing agent (Millipore, USA) was used to obtain the antigen-antibody complex band, and an image analyser (Bio-Rad, USA) was used to obtain band intensity via densitometric analysis. Protein expression was normalised to that of glyceraldehyde phosphatedehydrogenase (GAPDH) (Abcam, USA).

### Real-time PCR

Total ribonucleic acid (RNA) was extracted using TRIzol (Invitrogen, USA). After determining concentration and purity, the total RNA was reverse transcribed into complementary DNA (cDNA) using the High-Capacity cDNA Reverse Transcription Kit (Thermo Fisher, China) as per the manufacturer’s instructions. Real-time quantitative Polymerase Chain Reaction (PCR) was performed on a 2400 real-time PCR system using the SYBR® Green RT-PCR Reagents Kit (ABI, USA) to amplify the Fas, Fas-L and GAPDH genes. The Fas, Fas-L and GAPDH primer sequences used for amplification are shown in Table 1. Gene expression was normalized to that of GAPDH (Abcam, USA). Results were analyzed using the 2^-△△CT^ method, and fold change, as compared to control, was determined.

**Table 1:** Primers sequences used for real-time quantitative PCR

Genes	Forward primer	Reverse primer	Size (bp)
GAPDH	5′ 2AAATCGT2GCGTGACATTAA2 3’	5′ 2TCGTCATACTCCTGCTTG2 3’	381
FAS	5′2TCTAGTTGGAAAGAACCGAAGG2 3’	5′ 2TCTAGTTGGAAAGAACCGAAGG2 3’	306
Fas-L	5′2GGAATGGGAAGACACATATGGAACTGC2 3’	5′2CATATCTGGCCAGTAGTGCAGTAATTC2 3’	237

### Statistical analysis

Each experiment was performed at least three times. The values were expressed as mean ± standard deviation (SD). Data were evaluated using one-way analysis of variance (ANOVA), the post-hoc Test (Bonferroni and Tukey methods) was performed following ANOVA. SPSS version 20.0 (IBM Corp., Armonk, NY, USA) was used for the analyses. Values of *p* < 0.05 was considered to be statistically significant.

## Results

### HPLC analysis of SMS

**Fig. 1:**
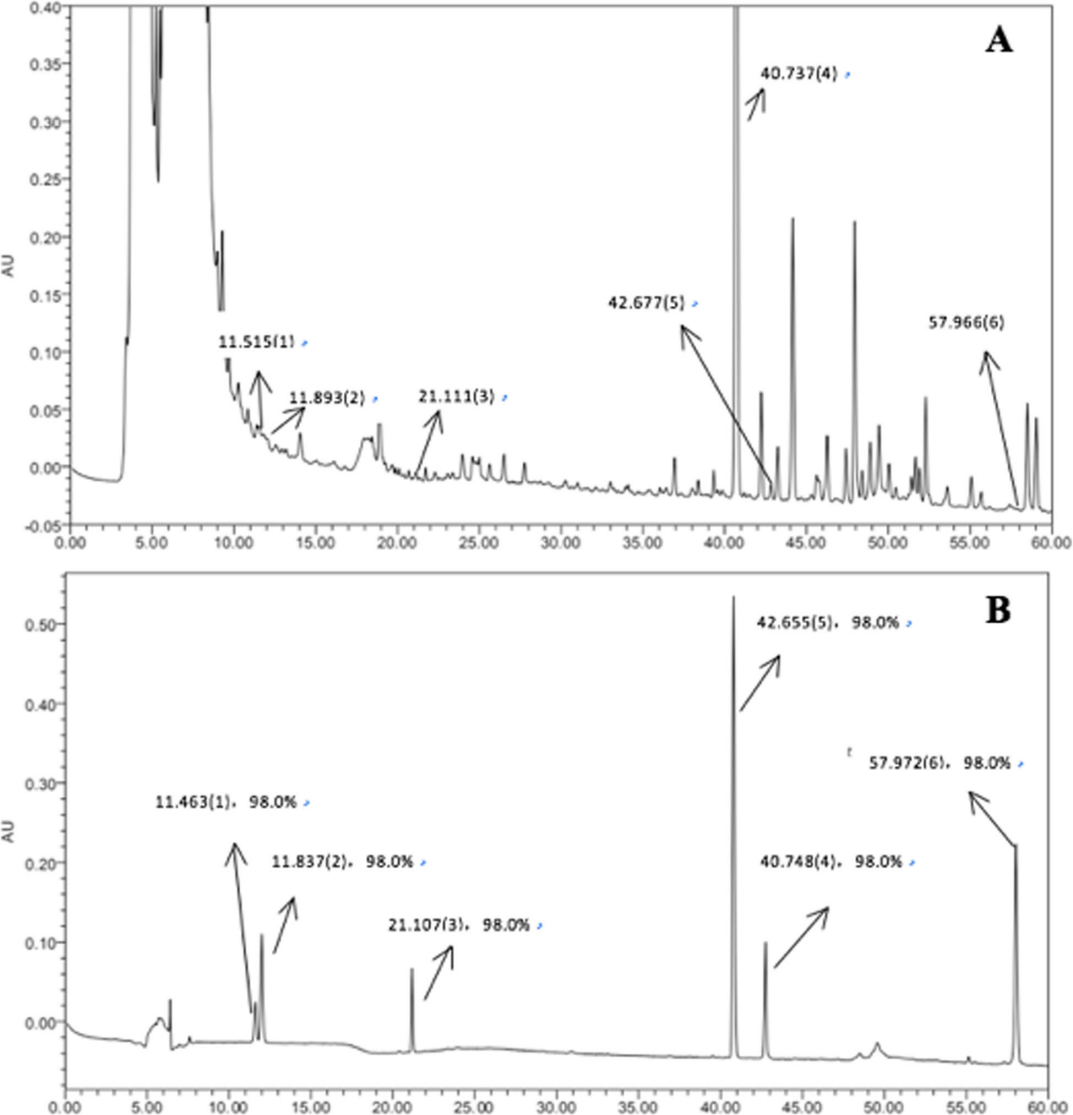
Chemical standardization of SMS (**a**) and its standard compounds (**b**) by high performance liquid chromatography (HPLC) fingerprint analysis (203 nm). In the HPLC fingerprint at an absorbance of 203 nm, the peaks corresponding to ginsenoside Re (1); ginsenoside Rg1 (2); ginsenoside Rb1 (3); schisandrin (4); ophiopogonin D (5); ruscogenin (6)

In order to standardise the chemical composition of the herbal medicine, we performed high performance liquid chromatography (HPLC) fingerprint analysis. Figure 1 shows a typical HPLC fingerprint of SMS, in which the major peaks were identified by comparing both the retention times of both SMS and the reference standards. Notably, 6 compounds in SMS, viz. 1) ginsenoside Re, 2) ginsenoside Rg1, 3) ginsenoside Rb1, 4) schisandrin, 5) ophiopogonin D, and 6) ruscogenin were properly identified.

### SMS increases cell viability within a certain dosage range

**Fig. 2:**
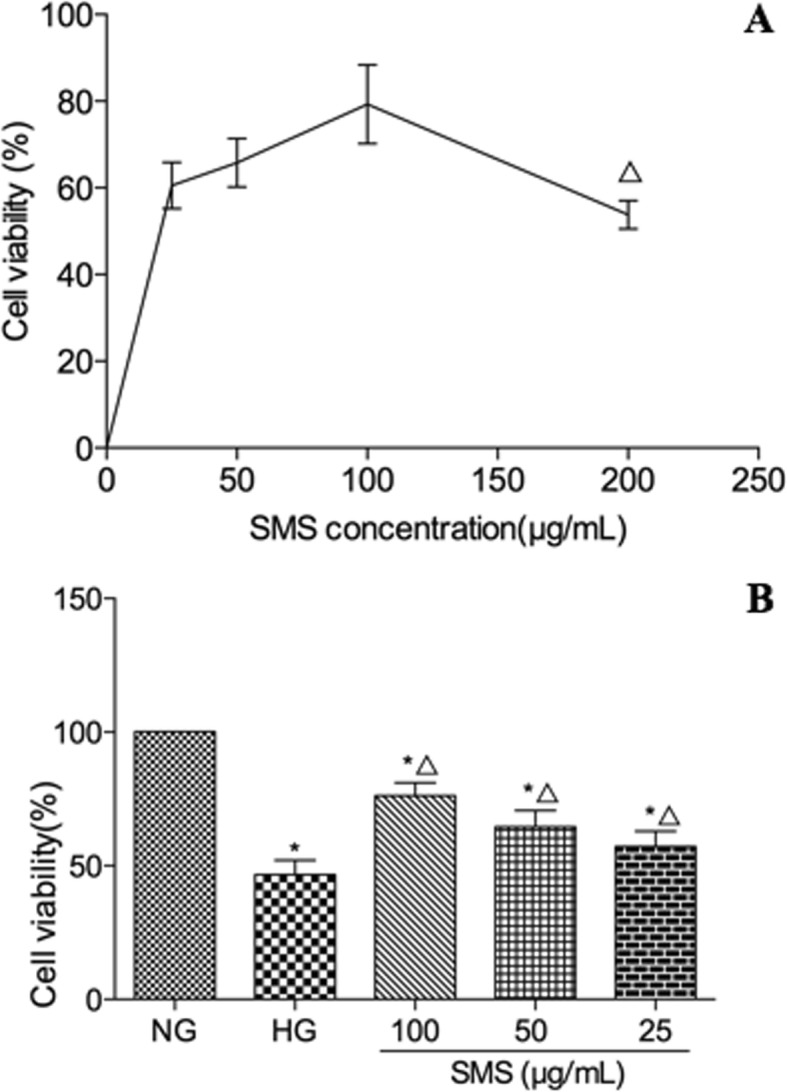
Effects of SMS on the H9C2 cell viability. **a** H9C2 cells were treated with different concentrations of SMS under normal glucose environment for 24 h. (△*p* < 0.05 vs. control cells). **b** SMS increases H9C2 cell viability exposed to HG (*n* = 3 in each group). (*p < 0.05: vs. NG group; △ *p* < 0.05: vs. HG group)

Hyperglycemia-induced H9C2 cells were treated with different concentrations of SMS for 24 h. The results revealed that H9C2 cell viability gradually increased (*p* < 0.05) with an increase in SMS concentrations. H9C2 cell viability was also observed to peak in the 100 μg/mL SMS group. For SMS concentrations reached above 200 μg/mL, H9C2 cell viability was observed to decline (*p* < 0.05; Fig. 2a). Although HG significantly reduced H9C2 cell viability compared to the NG group (46.7% ± 5.4% versus 76.2% ± 4.9%, *p* < 0.05), it appeared to be restored following an SMS pre-treatment. Compared with the HG group, SMS significantly increased H9C2 cell viability to 57.2% ± 5.7, 64.5% ± 6.2, and 76.2% ± 4.9% at concentrations of 25, 50, and 100 μg/mL (*p* < 0.05), respectively. Treatment with 100 μg/mL SMS significantly upregulated H9C2 cell viability compared with 25 μg/mL and 50 μg/mL SMS (p < 0.05), demonstrating a dose-dependent response (*p* < 0.05; Fig. 2b).

### SMS protects H9C2 cells from HG-induced apoptosis

**Fig. 3:**
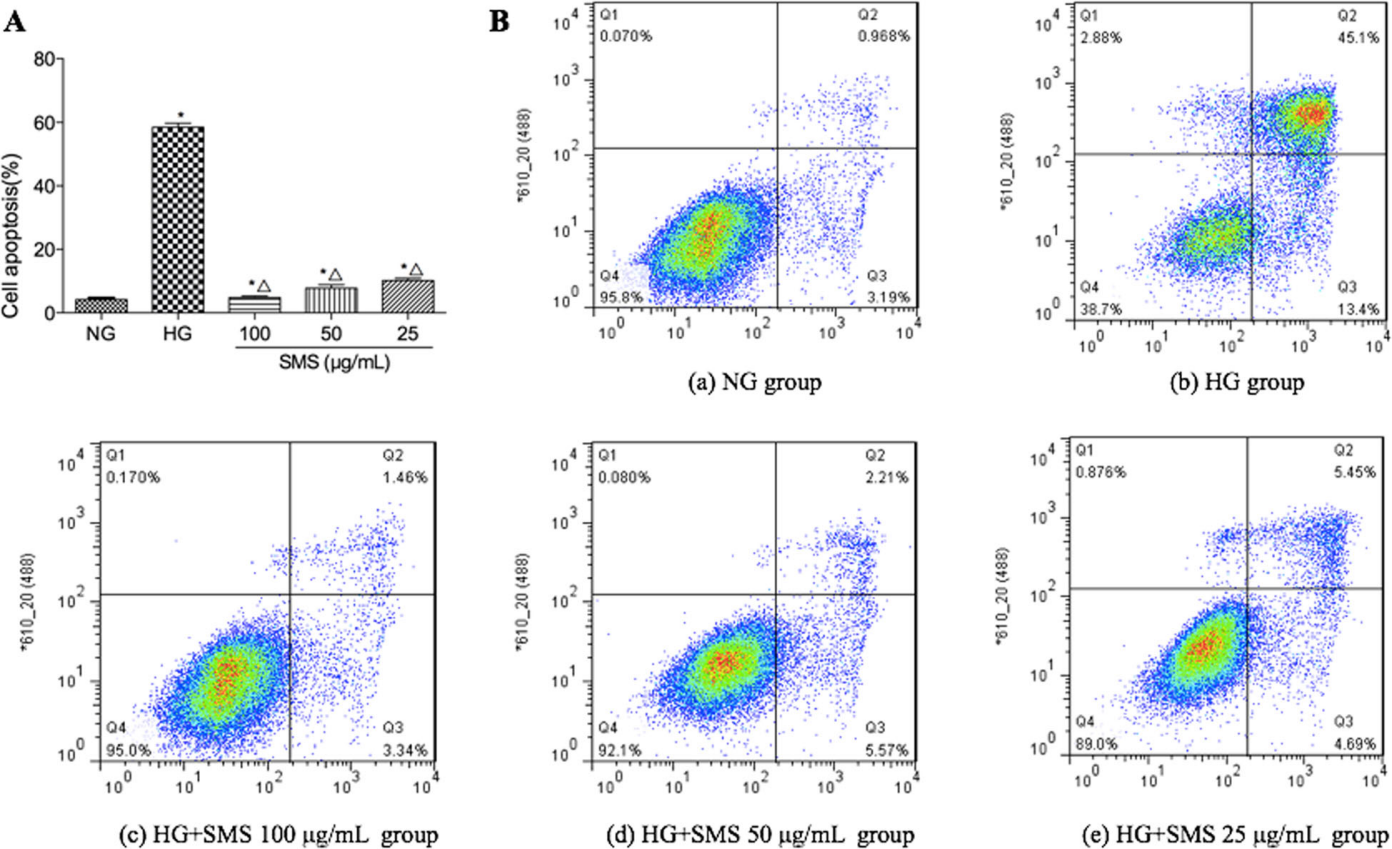
Effect of SMS on the H9C2 cell apoptosis exposed to HG. **a** The percentage of apoptotic cells in each group. **b** Cells were detected with a flow cytometer

Hyperglycemia significantly increased the incidence of apoptosis in H9C2 cells. As shown in Fig. 3 a and b, the percentage of apoptotic cells markedly increased in the HG group compared to the NG group (58.5% ± 1.29% versus 4.16% ± 0.64%, *p* < 0.05). However, compared with the HG group, SMS attenuated HG-induced apoptosis, and apoptotic cells markedly decreased to 10.14% ± 0.77, 7.78% ± 1.05, and 4.80% ± 0.52% at concentrations of 25, 50 and 100 μg/mL (*p* < 0.05), respectively. The proportion of apoptotic cells in 25, 50, and 100 μg/mL SMS-pretreated groups decreased with increasing concentration of SMS, which indicated to be significantly difference (*p* < 0.05) (Fig. 3).

### SMS affects the retention of the cell cycle in the H9C2 cells

**Fig. 4:**
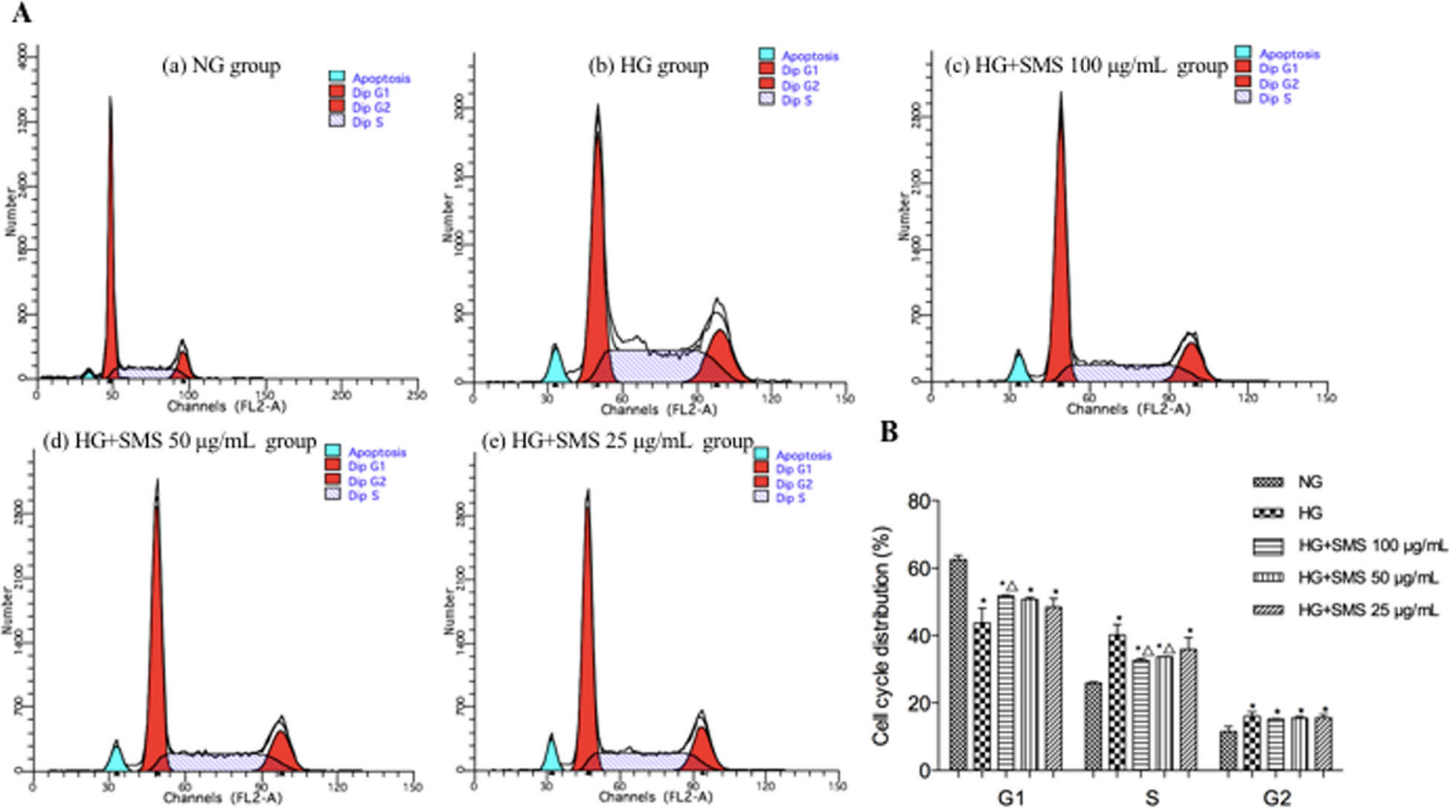
Effect of SMS on the cell cycle in H9C2 cells. **a** The cell cycle of each group in H9C2 cells. **b** Distribution of cell cycle for H9C2 cells after treated with various concentrations of SMS for 24 h. (*p < 0.05: vs. NG group; △ *p* < 0.05: vs. HG group)

To explore whether SMS-inhibited apoptosis was associated with cell cycle arrest, we detected the cell cycle distribution of H9C2 cells using flow cytometry to analyze cellular DNA content. As shown in Fig. 4, HG significantly reduced the percentage of H9C2 cells in the G1 phase compared with that in the NG group (43.77% ± 4.40% versus 62.58% ± 1.26%, *p* < 0.05). There was a marked increase in the percentage of cells in the G1 phase in H9C2 cells treated with 100 μg/mL of SMS for 24 h versus that in the HG group (51.78% ± 0.24% versus 43.77% ± 4.40%, *p* < 0.05), however, no significant difference existed between 25 and 50 μg/mL SMS-pretreated groups and HG group (51.78% ± 0.24% versus 43.77% ± 4.40, 50.76% ± 0.61% versus 43.77% ± 4.40%, respectively, for group 25 and 50, *p* > 0.05). Compared with the NG group, the percentage of H9C2 cells in the S phase significantly increased in the HG group (40.15% ± 3.13% versus 25.88% ± 0.30%, *p* < 0.05), whereas the percentage of cells in the S phase significantly decreased in H9C2 cells treated with 100 μg/mL and 50 μg/mL of SMS compared with that in the HG group (32.65% ± 0.52% versus 40.15% ± 3.13 and 33.71% ± 0.05% versus 40.15% ± 3.13%, respectively for group 100 and 50, *p* < 0.05). In addition, HG significantly increased the percentage of H9C2 cells in the G2 phase compared with that in the NG group (16.08% ± 1.26% versus 11.55% ± 1.56%, *p* < 0.05), but SMS-pretreated groups failed to significantly decrease the percentage of H9C2 cells compared with HG group (*p* > 0.05).

### SMS inhibits HG-induced apoptosis by decreasing p53 and Bax expression and increasing Bcl-2 expression

**Fig. 5:**
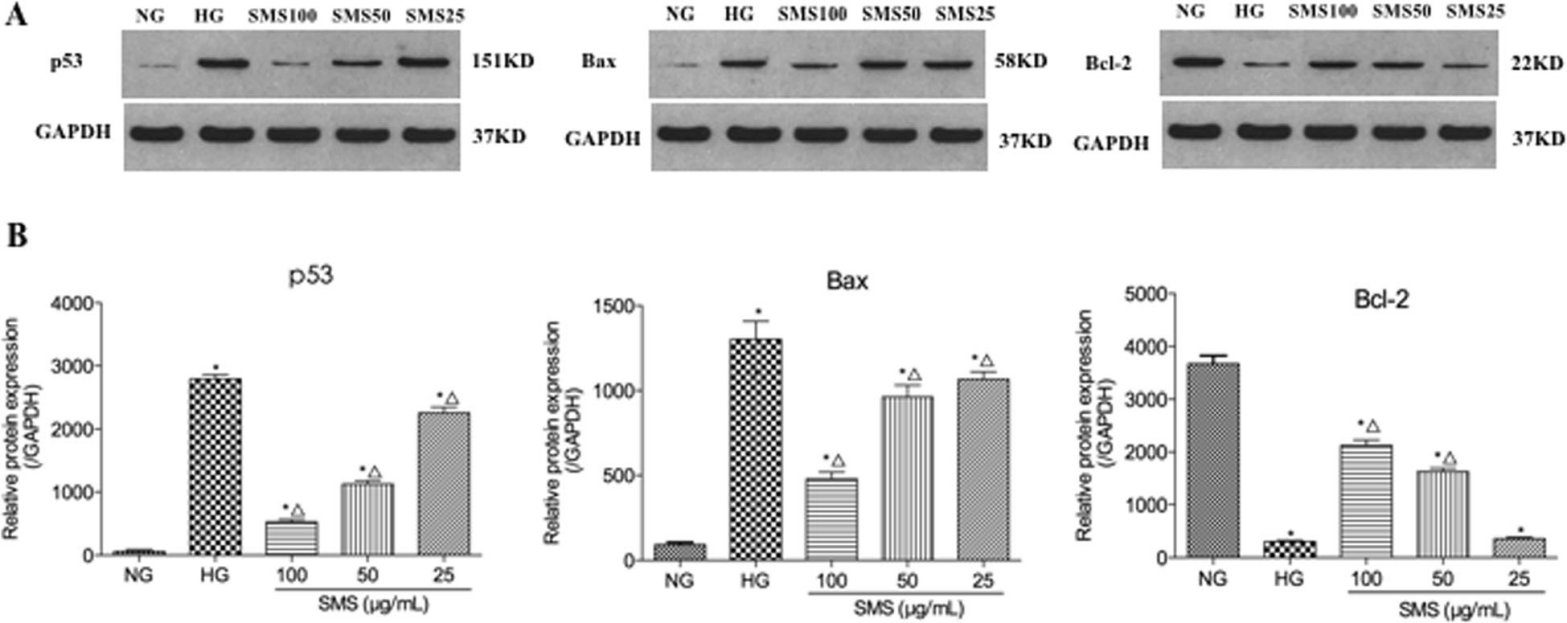
Western blot and quantitative measurement for p53, Bax and Bcl-2 protein expressions in H9C2 cells. **a** Western blot. **b** Quantitative measurement. (*p < 0.05: vs. NG group; △ *p* < 0.05: vs. HG group)

To determine the mechanisms underlying the SMS-mediated inhibition of HG-induced apoptosis, we examined apoptosis-related protein expression levels via western blotting. As presented in Fig. 5, Bax expression levels were markedly increased in the HG group compared to those in the NG group (1302.0 ± 106.1 versus 90.7 ± 13.9, *p* < 0.05). Bax expression level was decreased in a dose-dependent manner in the SMS pretreatment group relative to the H2O2 group to 1065.1 ± 44.1, 962.8 ± 67.3, and 479.9 ± 39.4 at concentrations of 25, 50 and 100 μg/mL (*p* < 0.05), and treatment with 100 μg/mL SMS significantly decreased Bax compared with 25 μg/mL and 50 μg/mL SMS (p < 0.05). Bcl-2 expression was also markedly decreased in the HG group when compared to that in the NG group (296.8 ± 22.1 versus 3668.0 ± 154.3, *p* < 0.05), and SMS treatment at 50 and 100 μg/mL for 24 h markedly increased Bcl-2 expression compared with HG group (1628.5 ± 64.6 versus 296.8 ± 22.1 and 2126.1 ± 100.9 versus 296.8 ± 22.1, respectively, *p* < 0.05). P53 expression levels were markedly increased in the HG group (2792.3 ± 70.2 versus 520.4 ± 41.8, *p* < 0.05). However, p53 expression levels was significantly reduced by SMS pretreatment in a concentration-dependent manner (2259.1 ± 89.2, 1126.5 ± 53.2, 520.4 ± 41.8, respectively, *p* < 0.05 for group 25, 50 and 100). Therefore, SMS inhibited HG-induced apoptosis probably by downregulating Bax expression and p53 expression and upregulating Bcl-2 expression.

### SMS downregulates Fas and FasL mRNA expression levels in H9C2 cells

**Fig. 6:**
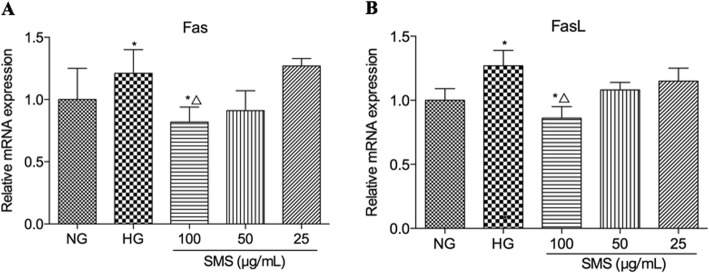
Real-time PCR analysis for Fas and FasLgene expression in H9C2 cells. **a** RT-PCR analysis for Fas. **b** RT-PCR analysis for FasL. (*p < 0.05: vs. NG group; △ *p* < 0.05: vs. HG group)

Fas and FasL gene expression levels were examined in order to further clarify the molecular mechanism of the SMS-mediated inhibition of apoptosis. RT-PCR results in Fig. 6a showed that Fas expression was significantly upregulated in the HG group (1.21 ± 0.19 versus 1.00 ± 0.25, *p* < 0.05), and this expression was dramatically reversed by SMS treatment at a concentration of 100 μg/mL for 24 h (0.82 ± 0.12 versus 1.21 ± 0.19, *p* < 0.05). However, no obvious difference in Fas expression was observed between the groups treated with SMS at 25 μg/mL and 50 μg/mL) and the HG group (*p* > 0.05). RT-PCR results in Fig. 6b showed that FasL expression was significantly upregulated in the HG group (1.27 ± 0.12 versus 1.00 ± 0.09, *p* < 0.05) and this expression was dramatically reversed by treatment with SMS at 100 μg/mL for 24 h (0.86 ± 0.09 versus 1.27 ± 0.12, *p* < 0.05). However, no significant difference in FasL expression was observed between the groups treated with SMS at 25 μg/mL and 50 μg/mL and the HG group (*p* > 0.05).

## Discussion

Sheng Mai San (SMS), an aqueous amalgamation of the extracts of *Radix Ginseng* (Ren Shen), *Radix Ophiopogonis* (Mai Dong), and *Fructus Schisandrae* (Wu Wei Zi), is a common traditional Chinese medicine (TCM) that is used to treat cardiovascular diseases [16–19, 23]. In the HPLC analysis, ginsenoside Re, ginsenoside Rg1, ginsenoside Rb1, schisandrin, ophiopogonin D, and ruscogenin were identified. Ginsenoside is an important active ingredient in *Radix Ginseng* (Ren Shen). Ginsenoside Re inhibits cardiomyocyte apoptosis by inhibiting expression of pro-apoptotic Bax gene and raising the ratio of Bcl-2/Bax [24]. Ginsenoside Rg1 inhibits the Jun N-terminal Kinase (JNK) pathway in H9c2 cells to protect against oxidative stress, which is regarded as a major cause of H9c2 cardiomyocyte apoptosis [25]. Ginsenoside Rb1 exerts significant and anti-diabetic effects by regulating the effects of glycolipid metabolism and improving insulin and leptin sensitivities [26]. Ginsenoside Rb1 protects HG- cardiomyocyte apoptosis, at least in part via the inhibition of caspase-3 activity and the Bax/Bcl-2 ratio [27]. Schisandrin B may attenuate the inflammatory response, oxidative stress and apoptosis in TSCI rats by inhibiting the p53 signaling pathway in rats [28]. Ophiopogonin D may protect cardiomyocytes against injury through suppressing endoplasmic reticulum stress [29]. The therapeutic effects of ruscogenin was determined in the Sheng-Mai-San. Although this is a traditional compound preparation, its constitution of bioactive components has been initially determined, including ginsenoside Rb1, ruscogenin and schisandrin. It exerts significant cardioprotection against myocardial ischemia injury by decreasing myocardium infarct size and regulating myocardial enzymes indexes [17]. In this study, the effect of SMS in the prevention and treatment of DCM was investigated. We showed that SMS treatment could inhibit HG-induced apoptosis and affect the retention of the H9C2 cell cycle. Notably, DM is associated with cardiac structural and functional changes, which lead to DCM.

The Bcl-2 protein family is the most widely studied and the most important apoptosis-regulating factor that regulates apoptosis mainly by maintaining the dynamic balance between pro-apoptotic and anti-apoptotic proteins [11]. P53 as a tumour suppressor gene that activates its downstream targets in a sequential manner in order to induce apoptosis and plays an important role in the prevention of cardiac fibrosis and heart failure [30, 31]. A previous study indicated that p53 directly activated Bax and suppressed Bcl-2 in order to permeabilize mitochondria and engage the apoptotic mechanism [32]. Our study investigated the expression levels of p53, Bax and Bcl-2 in HG-induced H9C2 cells. While p53 and Bax expression was perceptibly increased, Bcl-2 protein expression was notably decreased. This imbalance in protein expression was consistent with increased cardiomyocyte apoptosis. However, after SMS treatment, p53 and Bax expression was significantly downregulated and Bcl-2 expression was significantly upregulated when compared to the HG group.

To explore the molecular mechanisms underlying the SMS-mediated inhibition of HG-induced apoptosis in H9C2 cells, this study also investigated Fas and FasL expression. The Fas/FasL signaling pathway can directly trigger cardiomyocyte apoptosis and has been reported in studies related to cardiovascular diseases [33]. Fas is a transmembrane glycoprotein that is the most important death receptor for activation-induced cell death. On the contrary, Fas ligand (FasL) is a kind of type 2 transmembrane glycoprotein. Notably, the Fas/FasL combination causes an accumulation of intracellular ‘death-inducing signalling complexes’ that provide the necessary factors for Fas-mediated apoptosis [34]. This study indicated that Fas and FasL mRNA expression levels are significantly upregulated in the HG group; however, they are downregulated upon treatment with increasing concentrations of SMS, with the most significant downregulation observed at 100 μg/mL SMS. This suggests abatement in the apoptotic process by SMS.

The study has a few limitations that need to be addressed through further studies. First, the upstream mechanisms associated with the protective effect of SMS in cardiomyocyte apoptosis still need to be elucidated. Second, further experiments using a primary culture of neonatal rat cardiomyocytes are also needed to strengthen the conclusion that SMS inhibits apoptosis.

## Conclusion

In conclusion, SMS protected H9C2 cells from HG-induced damage and improved their viability by suppressing apoptosis. The protection offered by SMS against cardiomyocyte apoptosis was mediated by the downregulation of P53 expression and regulation of the Bcl-2/Bax signaling pathway. SMS at a 100 μg/mL concentration also downregulated Fas and FasL mRNA expression level in HG-induced H9C2 cells. We can, thus, surmise that SMS prevents and treats damage caused by DCM by regulating apoptosis-related mechanisms.

## Data Availability

The datasets used or analyzed during the study are available from the corresponding author on reasonable request.

## Abbreviations

Ang II: Angiotensin II
Annexin V-FITC/PI: Annexin-V fluorescein isothiocyanate/propidium iodide
ANOVA: One-way analysis of variance
Bax: Bcl-2 associated X protein
BCA: Bicinchoninic acid
Bcl-2: B cell lymphoma/leukemia-2
cDNA: complementary DNA
DCM: Diabetic cardiomyopathy
DM: Diabetes mellitus
DMEM: Dulbecco’s Modified Eagle Medium
DMSO: dimethylsulfoxide
Fas: Factor associated suicide
Fas-L: Factor associated suicide ligand
GAPDH: Glyceraldehyde phosphatedehydrogenase
HG: Hyperglycaemia
HPLC: High-performance liquid chromatography
JNK: Jun N-terminal Kinase
MTT: 3-(4,5-Dimethylthiazol-2-yl)-2,5-diphenyltetrazoliumbromide
NG: Normal glucose
p53: Protein 53
PBS: Phosphate buffered saline
PVDF: Polyvinylidene fluoride
RAS: Renin-angiotensin system
RIPA: Radio immunoprecipitation assay
RNA: Ribonucleic acid
SD: Standard deviation
SDS-PAGE: Sodium dodecyl sulfate-polyacrylaminde gel electrophoresis
TBS: Tris-buffered saline
TBST: Tris-Buffered Saline Tween-20
TCM: Traditional Chinese medicine
